# A comparison of activity demands between trial matches and in-season matches across multiple teams and seasons in semi-professional, male rugby league players

**DOI:** 10.5114/biolsport.2023.125586

**Published:** 2023-07-18

**Authors:** Thomas M. Doering, Nathan Elsworthy, Dean E. Callaghan, Ben Jones, Masaru Teramoto, Aaron T. Scanlan

**Affiliations:** 1 School of Health, Medical and Applied Sciences, Central Queensland University, Rockhampton, Australia; 2 Mackay Cutters Rugby League Club, Mackay, QLD, Australia; 3 Carnegie Applied Rugby Research (CARR) Centre, Carnegie School of Sport, Leeds Beckett University, Leeds, United Kingdom; 4 Leeds Rhinos Rugby League Club, Leeds, United Kingdom; 5 Division of Exercise Science and Sports Medicine, Department of Human Biology, Faculty of Health Sciences, the University of Cape Town and the Sports Science Institute of South Africa, Cape Town, South Africa; 6 England Performance Unit, Rugby Football League, Red Hall, Leeds, United Kingdom; 7 Department of Physical Medicine and Rehabilitation, University of Utah, Salt Lake City, UT, United States of America

**Keywords:** Acceleration, Deceleration, Football, Preparatory, Tackle, Team sports

## Abstract

Trial matches are frequently used for team preparation in rugby league competitions, making it essential to understand the demands experienced to assess their specificity to actual competition. Consequently, this study aimed to compare the activity demands between pre-season trial matches and early in-season rugby league matches. Following a repeated-measures observational design, 39 semi-professional, male rugby league players from two clubs were monitored using microsensors during two trial matches and the first two in-season matches across two consecutive seasons. Total distance, average speed, peak speed, absolute and relative high-speed running (HSR; > 18 km · h^-1^) and low-speed running (LSR; < 18 km · h^-1^) distance, as well as absolute and relative impacts, accelerations (total and high-intensity > 3 m · s^-2^), and decelerations (total and high-intensity < -3 m · s^-2^) were measured. Linear mixed models and Cohen’s d effect sizes were used to compare variables between match types. Playing duration was greater for in-season matches (*p* < 0.001, *d* = 0.64). Likewise, higher (*p* < 0.001, *d* = 0.45–0.70) activity volumes were evident during in-season matches indicated via total distance, HSR distance, LSR distance, total accelerations, high-intensity accelerations, total decelerations, and high-intensity decelerations. Regarding activity intensities, a higher average speed (*p* = 0.008, *d* = 0.31) and relative LSR distance (*p* = 0.005, *d* = 0.31) only were encountered during in-season matches. Despite players completing less volume, the average activity intensities and impact demands were mostly similar between trial and early in-season matches. These findings indicate trial matches might impose suitable activity stimuli to assist players in preparing for early in-season activity intensities.

## INTRODUCTION

Rugby league is one of the most popular team sports in Australia, with over 171,000 adults (> 15 years of age) and 133,000 children (< 14 years of age) participating in rugby league in 2020 [1]. Furthermore, over 5.7 million Australians aged > 14 years viewed televised matches of the 2020 National Rugby League (NRL) season [2]. The NRL represents the highest standard of male professional rugby league competition in Australia and New Zealand, and currently comprises 17 registered teams [3]. In turn, NRL teams have affiliations with semi-professional teams competing within state-wide competitions including the Queensland Cup, which are positioned directly beneath the NRL in the male player pathway [3]. Accordingly, many semi-professional, male rugby league players aim to develop their physical, technical, and mental attributes with ambition to transition to professional competition [4]. In this regard, semi-professional rugby league competitions not only offer a competitive, marketable product involving high-level players, but also play an important role in aiding the development of players to compete at professional levels [3]. To assist in player preparation and development it is important that semi-professional rugby league teams adopt strategies to ensure players are adequately prepared for match demands.

The monitoring of player demands using microsensors containing accelerometers and global positioning system (GPS) components, is increasingly adopted as routine practice within semi-professional rugby league teams [3]. This uptake of monitoring technology has enabled comparisons in match demands between semi-professional and professional rugby league players [5–7] and between successful and less successful semi-professional teams [8, 9]. Furthermore, player-worn microsensors permit a better understanding of the demands imposed on players during different training tasks and throughout match-play [10]. In this way, rugby league coaching staff can ensure their players are being exposed to appropriate demands during training to best prepare them for the rigours of competition.

The annual season in rugby league can be broadly split into pre-season, in-season, and off-season phases [11]. From a physiological perspective, the pre-season is a crucial phase whereby relevant fitness attributes can be developed through progressive overload of training stimuli to sufficiently enable players to cope with the demands of the upcoming in-season matches [11, 12]. In this regard, professional and semi-professional rugby league teams often participate in trial matches late in the pre-season, which are unofficial matches that do not determine official team standings. Coaching staff can use trial matches for various purposes including to evaluate new or development players, assess existing players in alternative positions, and rehearse set-plays alongside different player combinations. Nevertheless, given trial matches provide the most specific competitive context for rugby league teams during the pre-season, it is important to understand how the activity demands imposed during trial matches compare to in-season matches to ensure the observations made by coaching staff hold strong translation to official competition. For instance, if trial matches impose lower activity demands than in-season matches, the new players, player combinations, and set-plays that were rated positively by coaching staff during the trial matches may be less successful in competitive scenarios performed at higher intensities and/or for longer durations.

To date, only one study has compared the activity demands of trial matches to in-season matches in rugby league, examining a sample of professional male players [13]. Gabbett [13] reported professional, male rugby league players (n = 16) competing in the NRL had lower average speeds (*d* = 1.03–2.06), covered less distance at high (> 18 km · h^-1^; *d* = 0.26–0.98) and low speeds (< 18 km · h^-1^; *d* = 0.93–2.09), and had fewer repeated high-intensity efforts per minute (*d* = 0.90–2.99; all *p* < 0.05) in trial matches compared to in-season matches. These data show trial matches may not replicate key volumes or intensities of in-season matches in professional, male rugby league players, suggesting coaches may have difficulties in assessing whether players will be able to cope with the demands and team strategies will be successful during official competition based on observations made in trial matches. However, these findings cannot be simply extrapolated to semi-professional rugby league given the varied match demands reported between professional and semi-professional Australian rugby league competitions [5, 6, 13]. In this way, further research is needed to understand the demands of trial matches at the semi-professional level, which may have implications for player preparation processes. The purpose of this study was to compare the activity demands between trial matches and early in-season matches in semi-professional, male rugby league players.

## MATERIALS AND METHODS

### Experimental design

**FIG. 1:**
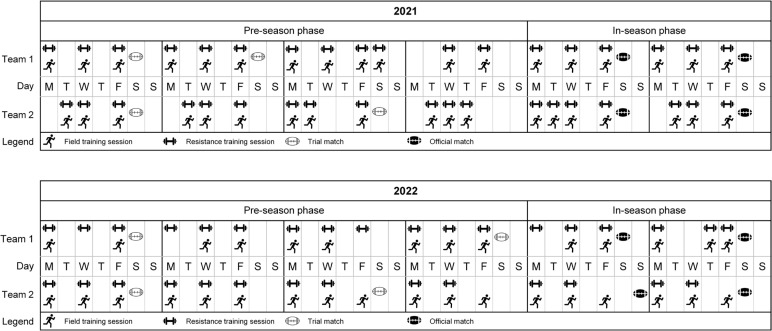
Schematic overview of training and match schedules for both participating semi-professional, male rugby league teams in the 2021 and 2022 seasons.

A repeated-measures observational study design was adopted whereby 60 semi-professional, male, rugby league players from two teams competing in the Queensland Cup were monitored during matches using microsensors throughout the 2021 and 2022 seasons. Each team participated in two trial matches in each season, towards the end of the pre-season phase. As a result, we monitored all available trial matches for these two teams across this two-year period. The first two matches of the in-season (i.e., round 1 and round 2) were also monitored for each team in each season to represent early in-season matches. Only the first two matches of the in-season phase were monitored to provide an equivalent comparison to trial matches while avoiding any temporal fluctuations in match demands that may occur with season progression [14]. A schematic timeline showing when the matches took place alongside the field- and gym-based training sessions leading into each match for each team are shown in Figure 1. The two teams participating in this study played each other in the first trial match in each season. Details of the matches monitored for both teams are shown in Table 1. Among the sample of games monitored, similar opponent rankings (mean opponent rank based on end of season ladder position for the respective year: trial matches = 10; in-season matches = 9), match times (mean start time [hh:mm]: trial matches = 18:10; in-season matches = 16:50), results (wins/losses/draws: trial matches = 4/4/0; in-season matches = 3/4/1), and identical distribution of match locations (home = 8; away = 8) were evident. All matches were 80 min in duration (plus stoppage time) in accordance with competition regulations.

**TABLE 1:** Details for the trial and in-season matches monitored.

Detail	2021 season				2022 season			
	*Trial matches*		*In-season matches*		*Trial matches*		*In-season matches*	
	*1*	*2*	*1*	*2*	*1*	*2*	*1*	*2*
***Team 1***								
Opponent rank*	12	8	4	9	11	12	14	12
Match location	Away	Home	Away	Home	Home	Away	Away	Home
Match time of day (hh:mm)	18:00	19:15	15:00	19:15	18:00	18:00	15:00	16:00
Match outcome	Loss	Win	Loss	Draw	Win	Win	Win	Win

***Team 2***								
Opponent rank*	14	7	5	3	5	10	12	13
Match location	Home	Home	Home	Away	Away	Away	Home	Away
Match time of day (hh:mm)	18:00	18:00	19:00	17:00	18:00	18:00	16:30	17:00
Match outcome	Win	Loss	Loss	Loss	Loss	Loss	Loss	Win

**Note**: * end of in-season ladder position used out of 14 teams

### Subjects

Monitored players were included in final analyses if they participated in at least three of the four monitored matches in at least one of the seasons, and registered at least 10 min of playing time in each match they played in. Therefore, a final sample of 39 players (age: 23.7 ± 1.9 years; height: 182.3 ± 5.4 cm; body mass: 96.4 ± 9.1 kg) were included in this study with 21 players excluded due to insufficient data being provided. This sample consisted of 20 forwards, 10 backs, and nine adjustables [15]. All players provided written informed consent after being made aware of the procedures, benefits, and risks associated with participation. Ethical approval was obtained from an Institutional Ethics Committee for use of the collected data across both teams (numbers: 0000023344 and 0000023985) in accordance with the standards of the Declaration of Helsinki.

### Procedures

All players were monitored during matches using Catapult PlayerTek™ GPS devices (PlayerTek™ Pod; Catapult Sports; Melbourne, Australia; specifications: 10-Hz sampling rate, 42 g mass, 84 mm × 42 mm × 21 mm dimensions), which were fitted in manufacturer-produced neoprene vests under playing jerseys ~15 min prior to pre-match warm-up. Data in Gaelic football players (n = 10) [16] support the validity (mean difference: distance vs. manual measurement of distance = < 0.01%, *p* > 0.05; peak speed vs. speed measured with a radar gun = 0.03%, *p* > 0.05) and retest reliability (coefficient of variation = 1.1% for total distance to 11.7% for very high-speed running distance at > 22 km · h^-1^) of PlayerTek™ devices. All collected data were downloaded from devices following each match and processed using proprietary software (PlayerTek™ Cloud; Catapult Sports; Melbourne, Australia). Match files for each player were trimmed to include only time when on the field and exclude any time when inter-changed out of matches and half-time breaks. Trimming of all match data files was performed using documented times and visual inspection of data traces in the proprietary software. Outcome variables taken from each match included total distance (m), average speed (m · min^-1^), as well as absolute (m) and relative (m · min^-1^) distance (i.e., average speed) performing low-speed running (LSR; < 18 km · h^-1^) and high-speed running (HSR; > 18 km · h^-1^) as commonly reported in the rugby league literature [15]. Furthermore, on-field duration (min), peak speed (m · s^-1^), absolute impacts (count; > 5 g), relative impacts (count · min^-1^), total accelerations (count; > 1 m · s^-2^), relative total accelerations (count · min^-1^), high-intensity accelerations (count; > 3 m · s^-2^), relative high-intensity accelerations (count · min^-1^), total decelerations (count; < -1 m · s^-2^), relative total decelerations (count · min^-1^), high-intensity decelerations (count; < -3 m · s^-2^), and relative high-intensity decelerations (count · min^-1^) were also measured in each match.

### Statistical analysis

Overall, 86 (49%) individual player samples for trial matches and 88 (51%) individual player samples for in-season matches were used for analyses, with 101 (58%) samples provided by Team 1 and 73 (42%) samples provided by Team 2. Linear mixed models were fitted to the data for each outcome variable. Specifically, player was entered as a random effect (n = 39) and match type was entered as a fixed effect (trial and in-season), while team (Team 1 and Team 2) was used as a covariate. Histograms developed for each outcome variable according to match type revealed that most were normally distributed, with some possessing slightly skewed distributions. No outliers were identified in any outcome variable. Accordingly, to obtain robust models, each linear mixed model was constructed by obtaining the robust estimate of variance (Huber-White sandwich estimator) [17, 18]. An unstandardized regression coefficient (β) and its 95% confidence intervals (CI) along with the marginal means and standard errors (SE) for each match type [19] were calculated for each model. Linear mixed models have been advocated for comparisons in monitoring variables within applied sport science observational studies involving repeated measurements on players [20] and have been readily used to compare demands placed on players across different seasonal phases in team sports [21–24]. An alpha level was set at 0.05 to indicate statistical significance. Further, Cohen’s *d* was computed for each model from values and the pooled standard deviation for each outcome variable [25], with the following interpretations applied: *trivial* = < 0.20; *small* = 0.20–0.59; *moderate* = 0.60–1.19; *large* = 1.20–1.99; *very large* = 2.00–3.99; and *extremely large* = > 4.00 [26]. All analyses were conducted using Stata/MP (version 17.0 for Windows; StataCorp LLC; College Station, TX, USA).

## RESULTS

**FIG. 2:**
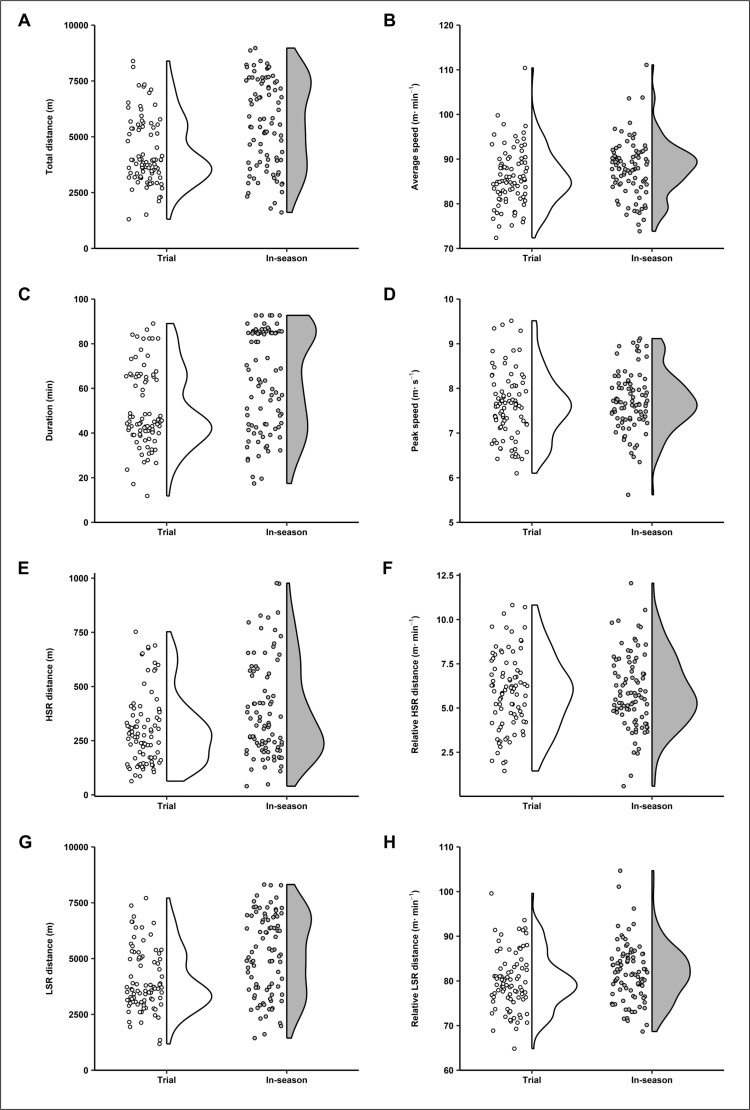
Individual data points alongside half-violin plots for (A) total distance, (B) average speed, (C) duration, (D) peak speed, (E) high-speed running (HSR) distance, (F) relative HSR distance, (G) low-speed running (LSR) distance, and (H) relative LSR distance during trial and in-season matches among semi-professional, male rugby league players (n = 39).

**FIG. 3:**
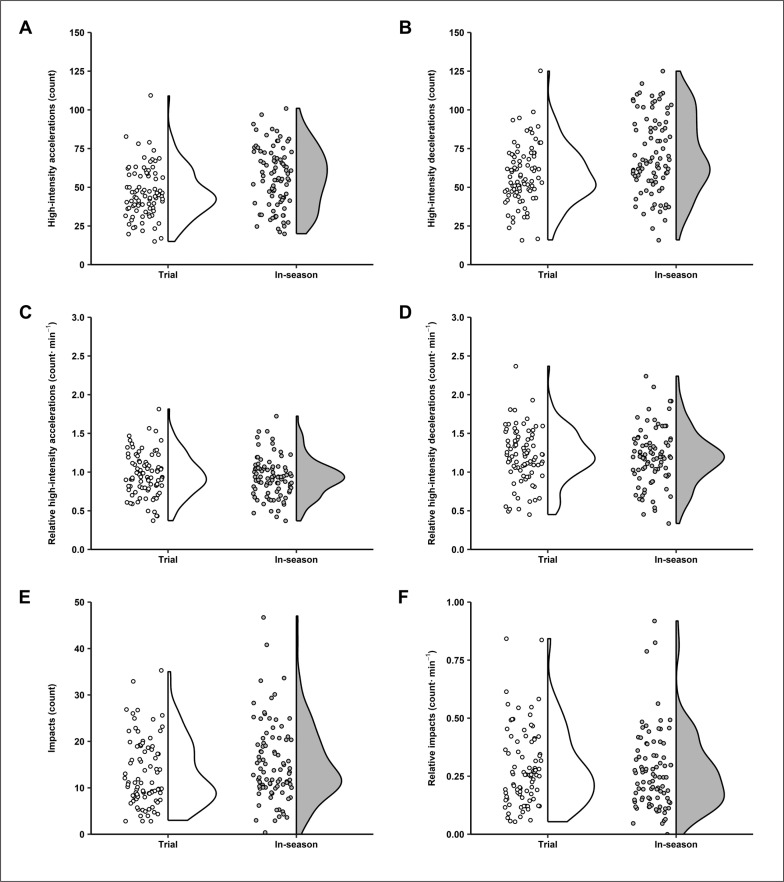
Individual data points alongside half-violin plots for (A) high-intensity accelerations, (B) high-intensity decelerations, (C) relative high-intensity accelerations, (D) relative high-intensity decelerations, (E) impacts, and (F) relative impacts during trial and in-season matches among semi-professional, male rugby league players (n = 39).

Results from the linear mixed model analyses including comparison statistics between trial matches and in-season matches are shown in Table 2. Furthermore, the individual data points for each outcome variable encompassing both seasons and both teams are shown in Figures 2 and 3. Statistically significant (*p* < 0.05) differences between match types were apparent for several variables. Specifically, players spent significantly more time on-field during in-season matches compared to trial matches (*moderate*), and thus attained significantly higher absolute outputs for total distance (*moderate*), HSR distance (*small*), LSR distance (*moderate*), accelerations (*moderate*), high-intensity accelerations (*small*), decelerations (*moderate*), and high-intensity decelerations (*small*). Players also attained a significantly higher average speed (*small*) and relative LSR distance (*small*) during in-season matches compared to trial matches. In contrast, non-significant (*p* > 0.05) differences were evident between match types for relative HSR distance (*trivial*), peak speed (*trivial*), total impacts (*small*), relative impacts (*trivial*), relative accelerations (*trivial*), relative high-intensity accelerations (*trivial*), relative decelerations (*trivial*), and relative high-intensity decelerations (*trivial*). Team, which was entered as a covariate, was not significant to any model (*p* > 0.05).

**TABLE 2:** Comparison of activity demands between trial and in-season matches in semi-professional, male, rugby league players (n = 39).

Outcome variable	Marginal mean ± SE		Statistical comparison		
	*Trial*	*In-season*	*β (95% CI)*	*p*	*d, interpretation*
Playing duration (min)	50.3 ± 2.2	63.1 ± 3.5	12.8 (8.2, 17.5)	< 0.001*	0.64, *moderate*
Total distance (m)	4266 ± 193	5477 ± 308	1,211 (812, 1,610)	< 0.001*	0.69, *moderate*
Average speed (m · min^-1^)	85.5 ± 0.8	87.5 ± 0.9	1.9 (0.5, 3.4)	0.008*	0.31, *small*
HSR distance (m)	306 ± 22	392 ± 34	86 (43, 129)	< 0.001*	0.45, *small*
Relative HSR distance (m · min^-1^)	6.02 ± 0.30	6.12 ± 0.30	0.09 (-0.42, 0.61)	0.72	0.04, *trivial*
LSR distance (m)	3957 ± 177	5086 ± 283	1,129 (759, 1,500)	< 0.001*	0.70, *moderate*
Relative LSR distance (m · min^-1^)	79.4 ± 0.9	81.3 ± 1.0	2.0 (0.6, 3.2)	0.005*	0.31, *small*
Peak speed (m · s^-1^)	7.63 ± 0.11	7.75 ± 0.09	0.12 (-0.03, 0.26)	0.11	0.17, *trivial*
Impacts (count)	13.3 ± 0.9	15.2 ± 1.2	2.0 (-0.1, 4.0)	0.06	0.25, *small*
Relative impacts (count · min^-1^)	0.29 ± 0.02	0.26 ± 0.02	-0.02 (-0.06, 0.02)	0.27	-0.13, *trivial*
Accelerations (count)	210 ± 9	265 ± 15	55 (35, 75)	< 0.001*	0.65, *moderate*
Relative accelerations (count · min^-1^)	4.32 ± 0.11	4.30 ± 0.12	-0.02 (-0.15, 0.12)	0.80	-0.02, *trivial*
High-intensity accelerations (count)	45.5 ± 2.0	54.6 ± 2.8	9.1 (4.6, 13.6)	< 0.001*	0.51, *small*
Relative high-intensity accelerations (count · min^-1^)	0.95 ± 0.04	0.91 ± 0.04	-0.04 (-0.09, 0.01)	0.15	-0.15, *trivial*
Decelerations (count)	205 ± 9	260 ± 14	55 (35, 75)	< 0.001*	0.67, *moderate*
Relative decelerations (count · min^-1^)	4.20 ± 0.10	4.21 ± 0.11	0.01 (-0.12, 0.14)	0.87	0.02, *trivial*
High-intensity decelerations (count)	56.7 ± 2.5	69.2 ± 3.8	12.5 (7.0, 18.0)	< 0.001*	0.56, *small*
Relative high-intensity decelerations (count · min^-1^)	1.19 ± 0.05	1.17 ± 0.06	-0.03 (-0.09, 0.03)	0.41	-0.07, *trivial*

***Note***: Comparisons are presented as in-season match vs. trial match (reference category). Player entered as random effect and match type entered as fixed effect with team used as a covariate in linear mixed models.

* denotes significant difference with alpha set at < 0.05.

***Abbreviations***: SE = standard error; β = unstandardized regression coefficient; CI = confidence intervals; HSR = High speed running (> 18 km · h^-1^); LSR = low speed running (< 18 km · h^-1^).

## DISCUSSION

The aim of this study was to compare activity demands between trial matches and in-season matches in male rugby league players from two semi-professional Australian teams across two consecutive seasons. Our findings show players worked at similar peak (i.e., peak speed) and average intensities (for all outcome variables except average speed and relative LSR distance) in trial and in-season matches but lower activity volumes (for all outcome variables except impacts) were achieved in trial matches. These findings add to the limited evidence base outlining the activity demands imposed during trial matches compared to in-season matches in rugby league [13] and in wider team sports [27–29].

Our results provided for semi-professional rugby league players indicate pre-season trial matches induce similar activity intensities for most outcome variables, compared to early in-season matches. Specifically, peak speed, relative HSR distance, and relative total and high-intensity accelerations and decelerations were comparable (*p* > 0.05) across match types. These findings partly contrast those reported in professional, male rugby league players [13]. Specifically, Gabbett [13] showed players covered less (*p* < 0.05) relative HSR distance (> 18 km · h^-1^) during trial compared to in-season matches. Reasons for variation in findings between our study and those previously reported [13] might be attributed to the different motivations underpinning performance during trial matches as well as psychological profiles [30] between male rugby league players competing at different playing levels. However, it is important to note that the previous study [13] monitored four trial matches and 13 in-season matches across multiple months, whereas we specifically monitored 2 trial matches and the first 2 in-season matches for each team across an acute 6-week period. Indeed, relative HSR distance [14] has been shown to increase during middle (matches 9–16) and late (matches 17–24) phases of the in-season compared to the early in-season phase (matches 1–8) in professional, male rugby league players. Consequently, the inclusion of matches beyond the early in-season phase in the previous study [13] may have augmented the in-season HSR demands relative to trial matches. Nevertheless, similar to the findings of Gabbett [13], our findings show that in-season matches are played at a higher (*p* < 0.05) average speed (m · min^-1^) than trial matches. However, practically speaking, the differences in average speed in our study were small (*d* = 0.31; 85.5 ± 0.8 vs 87.5 ± 0.9 m · min^-1^), compared to the large differences reported by Gabbett [13] (*d* = 1.03–2.06). Consequently, the collective evidence suggests players move at slower speeds during trial matches compared with in-season matches when not engaged in high-intensity passages of play, but these differences may be of smaller magnitude at the semi-professional level. These findings may be attributed to factors that promote more stationary and walking activity between high-speed running bouts in trial matches, such as the lower fitness capacities typically evident among players in the pre-season compared to mid-season [31]. Moreover, poorer skill execution due to less experienced players being trialled in the pre-season, or due to less cohesive team dynamics because of unfamiliar player combinations, may have promoted greater error rates in general play leading to more stoppages.

The comparable data we observed for peak speed and most relative activity demand variables reported per minute of play between trial and in-season matches has important implications for player preparation strategies in semi-professional rugby league teams. Although coaches likely adopt strategies that restrict the playing time of some players to evaluate a broader range of players within the squad during trial matches, it is essential that players are exposed to intensities akin to in-season matches when on the field. In this way, the observations made by coaching staff during trial matches will potentially carry stronger translation to in-season match-play if players demonstrate they can repeatedly execute fundamental activities and team strategies are effective in scenarios performed at intensities specific to official competition. Furthermore, given exercise intensity is an important mediator in acutely enhancing mitochondrial adaptations [32], repeated-sprint ability [33], and aerobic capacity [32, 34], our observations suggest trial matches may partly elicit suitable stimuli to promote physiological adaptations specific to in-season matches at the semi-professional level.

In line with the comparisons made for peak speed and most relative ( · min^-1^) outcome variables, total impacts (count) and impacts per minute were comparable across match types in our study. These findings partly align with previous data reported in professional, male rugby league players [13] showing a similar number of (*p* > 0.05) total impacts (for adjustable and outside back positions) and impacts per minute (for forward, adjustable, and outside back positions) were encountered in trial and in-season matches. We speculate that poorer tackling proficiency may have contributed to comparable impact metrics between match types, despite lower average playing durations in trial matches. For instance, poorer tackling ability (effect size = -0.24) has been observed in the pre-season compared to in-season [35], and repeated high-intensity efforts (i.e., sprints, tackling, and jogging) inducing fatigued states have been shown to diminish (effect size = -1.17) tackling ability [36] in semi-professional, male rugby league players. Consequently, lower tackling proficiencies and greater susceptibility to fatigue in the pre-season may lead to more missed tackles and faster opposition play-the-ball speeds, increasing tackling requirements during trial matches. However, outcome variables indicative of physical fitness and technical abilities were not measured in our study and should be included in future research on this topic to confirm these suppositions.

While the activity intensity data we collected yields some important insights, it should be interpreted alongside activity volume data to comprehensively understand the match stimuli encountered in trial matches and in-season matches. In our study, players underwent significantly greater playing durations during in-season matches compared to trial matches, which underpin the significantly greater activity volumes observed during in-season matches in the form of total, HSR, and LSR distances, as well as accelerations and decelerations. In this regard, a longer exposure to match-play provides increased opportunity for players to participate in more match tasks producing greater field coverage and changes in movement speed and direction, elevating total distance and acceleration variables. The reduced match exposure during trial matches is likely due to various coaching strategies employed in the pre-season, including trialling different players across positions for selection and limiting the match exposure for key players to minimise injury risk. These strategies are possible in trial matches given the unrestricted number of players able to be selected for matches and interchanges available during matches. Indeed, these patterns in match exposure, total distance, and HSR distance mirror those observed in professional rugby league [13] and other team sports such as Australian rules football [28], suggesting mismatches in activity volumes are common between trial matches and in-season matches in team sports. Given consistent training schedules were administered in weeks when trial matches and in-season matches were played in our study, the lower match activity volumes may not adequately prepare players for the overall weekly stimuli faced during the early in-season phase, but a detailed analysis of session and accumulated training load was outside the scope of this investigation.

Some limitations should be acknowledged when interpreting our findings. First, reported data are representative of two semi-professional, Australian, male rugby league teams and may not be representative of all semi-professional teams, teams competing at other playing levels, or female competitions. Second, although data support the validity (distance and peak speed) and reliability (distance and HSR distance) of running metrics derived from the PlayerTek™ devices [16] used for monitoring players in our study, accelerometer-derived variables are yet to be independently validated or tested for reliability. Third, despite the multi-season sample of included matches in our study being well-matched for wins and losses between match types, we acknowledge that differences in stoppages within play may influence mean intensity-related activity demands. Furthermore, despite conducting a multi-team study, we were unable to conduct analyses according to playing position due to an insufficient sample size. Finally, the perceptual and cardiovascular demands, as well as the technical and tactical demands encountered by players were not recorded in our study and may show useful differences between trial matches and in-season matches. Consequently, wider research on this topic is encouraged examining other teams, using additional outcome variables, and conducting positional analyses.

## CONCLUSIONS

This study is the first to quantify and compare activity demands between trial matches and in-season matches in semi-professional, male rugby league players, providing important insights relevant to player preparation at this level of play. The activity intensities for most variables (i.e., peak speed, relative HSR distance, relative impacts, relative total and high-intensity accelerations and decelerations) and total impacts attained during trial matches were comparable to those attained during official matches held early in the in-season. In contrast, the activity volumes in in-season matches significantly exceeded those encountered during trial matches; the average speed and relative LSR distance in in-season matches were also higher than trial matches, but the differences were small (*d* = 0.31). These findings hold important implication to player preparation strategies for semi-professional rugby league teams. Specifically, given the importance of exercise intensity in facilitating positive aerobic adaptations in team sport players [37], the comparable activity intensities between match types in our study suggest trial matches may be useful for coaching staff to physically prepare semi-professional rugby league players for in-season match-play. Moreover, the impact exposure encountered during trial matches may also adequately replicate the demand during early in-season matches. However, the lower playing time and activity volumes during trial matches suggests players may not be adequately prepared for the entire in-season match stimuli without additional volume overload. Consequently, elevated match volume demands encountered in the early in-season may increase risk of maladaptive responses and injury, particularly for players expected to play entire matches.
